# Shared and Non-Shared sIgA-Coated and -Uncoated Bacteria in Intestine of Mother–Infant Pairs

**DOI:** 10.3390/ijms23179873

**Published:** 2022-08-30

**Authors:** Mengfan Ding, Haiqin Chen, Renqiang Yu, Reynolds Paul Ross, Catherine Stanton, Hao Zhang, Bo Yang, Wei Chen

**Affiliations:** 1 State Key Laboratory of Food Science and Technology, Jiangnan University, Wuxi 214122, China; 2 School of Food Science and Technology, Jiangnan University, Wuxi 214122, China; 3 Department of Neonatology, The Affiliated Wuxi Maternity and Child Health Care Hospital of Nanjing Medical University, Wuxi 214122, China; 4 International Joint Research Center for Probiotics & Gut Health, Jiangnan University, Wuxi 214122, China; 5 APC Microbiome Ireland, University College Cork, T12 K8AF Cork, Ireland; 6 Teagasc Food Research Centre, Moorepark, Fermoy, P61 C996 Co Cork, Ireland; 7 National Engineering Research Center for Functional Food, Jiangnan University, Wuxi 214122, China; 8 Wuxi Translational Medicine Research Center and Jiangsu Translational Medicine Research Institute Wuxi Branch, Wuxi 214122, China

**Keywords:** sIgA-coated bacteria, sIgA-uncoated bacteria, bifidobacterial community

## Abstract

The infant gut microbiota is critical for promoting and maintaining early-life health. The study aimed to analyze the composition of sIgA-coated and sIgA-uncoated bacterial communities at genus level and lactobacilli and bifidobacterial communities at species level in human breast milk (HBM) and infant and maternal feces. Eleven pregnant women were recruited successfully. HBM; infant feces during colostrum, transition, and mature stages; and maternal feces within the mature stage were collected. sIgA-coated and sIgA-uncoated bacteria were separated with magnetic-activated cell sorting. Then, 16S rRNA sequencing, bifidobacterial *groEL* gene sequencing, and lactobacilli *groEL* gene sequencing were performed to analyze the bacterial community. PCoA revealed that the compositions of sIgA-coated and sIgA-uncoated bacteria were different among HBM and infant and maternal feces. Higher relative abundance of sIgA-uncoated *Bifidobacterium* was found in the three lactation stages in infant feces compared to the corresponding HBM, and a higher relative abundance of sIgA-uncoated *Faecalibacterium* was found in maternal feces compared to HBM and infant feces. For bifidobacterial community, sIgA-coated and sIgA-uncoated *B. longum* subsp. *infantis* and *B. pseudocatenulatum* was dominant in infant feces and maternal feces, respectively. The relative abundance of sIgA-uncoated *B. longum* subsp. *infantis* was significantly higher in infant feces compared to that in maternal feces. For the *Lactobacillus* community, *L*. *paragasseri* and *L*. *mucosae* were dominant in infant and maternal feces, respectively. HBM and infant and maternal feces showed distinct diversity and composition of both sIgA-coated and sIgA-uncoated bacteria at genus level. Infant and maternal feces showed similar composition of *Bifidobacterium* at species level. The same *Bifidobacterium* species could be detected both in sIgA-coated and -uncoated form. This article provided deeper understanding on the microbiota profile in HBM and infant and maternal feces.

## 1. Introduction

The composition of the infant gut microbiota is a key factor influencing host immune system development and maturity [1]. Research to date indicates that the gut microbiota in early life is derived mainly from the maternal gastrointestinal tract and breast milk [2,3]. Certain bacteria in the intestine can become coated with secretory immunoglobulin A (sIgA), and after binding to sIgA, microbiota in the maternal intestine may specifically adhere to microfold cells (M cells) [4], which are then transported from the intestinal lumen to sub-epithelial dendritic cells and can enter the mammary gland through the entero-mammary pathway and colonize the infant’s gut through consuming breast milk [5,6]. Bacteria coated by sIgA may have a colonization advantage within the gut mucosal surface by excluding exogenous competitors [7].

The structure and dynamics of sIgA-coated bacteria in the gut can be a biomarker to predict the occurrence of disease [8,9]. For example, infants suffering from necrotizing enterocolitis showed a lower relative abundance of sIgA-coated bacteria, such as Bifidobacteriaceae and Prevotellaceae, and a higher relative abundance of sIgA-coated Enterobacteriaceae [10]. In adults with inflammatory bowel disease, the ratio of sIgA-coated bacteria in feces was increased compared with healthy controls [11]. Additionally, at 12 months of age, the relative abundances of sIgA-coated bacteria in children with allergy symptoms, particularly asthma, were lower than in healthy children: *Faecalibacterium* and *Bacteroides* were in sIgA-coated form in healthy children at 1 month and 12 months, whereas these two genera were uncoated in children with allergic manifestations [12]. Therefore, it is necessary to summarize the composition of sIgA-coated bacteria in the gastrointestinal tract of healthy infants for disease prediction.

For the detection of sIgA-coated bacteria, the methods mainly used include flow cytometry, magnetic-activated cell sorting (MACS), and a combination of the two approaches to improve the accuracy [9]. Previous studies have already reported that *Bifidobacterium*, *Staphylococcus*, *Lactobacillus*, *Escherichia*/*Shigella*, *Clostridium*, and *Bacteroides* can be detected in the sIgA-coated form in the infant intestine from 1 month to 12 months old [12,13,14]. In the adult intestine, *Actinomyces*, *Bifidobacterium*, *Ruminococcus*, *Akkermansia*, *Bacteroides*, *Roseburia*, *Paraprevotella*, and *Dorea* were detected in the sIgA-coated form [9,15,16]. However, little research has focused on the profile of sIgA-coated bacteria in human breast milk. More than 40% of the bacteria in human milk are coated with sIgA, according to one report [17], and another study found that *Bifidobacterium*, *Lactobacillus*, and *Streptococcus* were detected in the sIgA-coated form in human breast milk [18]. In addition, the existing research has mainly focused on the composition of sIgA-coated bacteria and did not compare the sIgA-coated bacteria and sIgA-uncoated bacteria at the genus level and species level of *Bifidobacterium* and *Lactobacillus*.

Hence, the current study aimed to analyze the composition of sIgA-coated bacteria and sIgA-uncoated bacteria in breast milk, infant feces, and maternal feces at genus level and at species level among *Bifidobacterium* and *Lactobacillus*. Within the study, 16S rRNA sequencing, bifidobacterial *groEL* sequencing, and lactobacilli *groEL* sequencing, coupled with MACS, were used to separate and analyze the composition of sIgA-coated and -uncoated bacteria.

## 2. Results

### 2.1. Total Bacterial Structure in HBM and Maternal and Infant Feces

**Figure 1:**
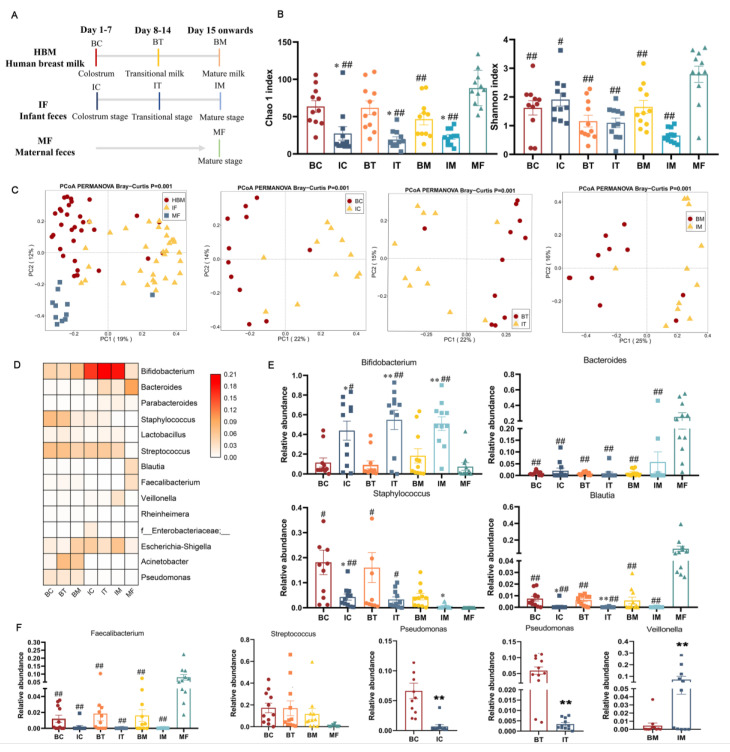
Diversity and composition of total bacteria in HBM and infant and maternal feces. (**A**) Sample collection type and time point. (**B**) Alpha diversity. (**C**) Beta diversity. PERMANOVA was used to calculate differences among samples based on Bray–Curtis distance. (**D**) Heatmap of total bacterial composition in HBM and infant and maternal feces. Genera of relative abundances > 0.01% and calculated with log10 are presented. The darker color corresponds to higher relative abundance. (**E**,**F**) Significant difference of genera between different samples. *, *p* < 0.05; **, *p* < 0.01three stages of infant feces compared to BC, BT, and BM. #, *p* < 0.05; ##, *p* < 0.01, three stages of HBM and infant feces compared to maternal feces. HBM, IF, and MF stand for group human breastmilk and infant and maternal feces. BC, BT, BM, IC, IT, and IM stand for colostrum, transitional milk, mature milk, and infant feces corresponding to HBM stages.

Eleven mother–infant pairs were recruited who delivered at the Affiliated Wuxi Maternity and Child Health Care Hospital of Nanjing Medical University from February 2020 to April 2021 (Ethics number: 2020-01-0302-03). All the samples underwent 16S rRNA amplicon sequencing. The sample collection type and time point were showed in Figure 1A. Chao1 and Shannon indexes were used to assess the alpha diversity of bacterial community, respectively (Figure 1B). Maternal feces (MF) showed significantly higher richness compared with all the three stages of infant feces (IC, the stage corresponding to colostrum; IT, the stage corresponding to transition milk; IM, the stage corresponding to mature milk; *p* < 0.01 for all) and was only significantly higher than mature milk (BM, *p* < 0.01) but not transition milk (BT) or colostrum milk (BC). Additionally, the richness of those three stages of infant feces (IC, IT, IM) was significantly lower than the corresponding human breast milk (BC, colostrum; BT, transitional milk; *p* < 0.05 for all). Similar to the richness of bacteria, the diversity of the MF group was significantly higher than that of IC (*p* < 0.05), IT (*p* < 0.01) and IM (*p* < 0.01) and also significantly higher than that of BC, BT, and BM (*p* < 0.01), whereas no significant difference in richness was found among infant feces and human breast milk at any stage (Figure 1A). Principal coordinate analysis (PCoA) was used to represent the beta diversity based on Bray–Curtis distance matrices. The composition of bacteria in HBM, maternal feces, and infant feces was significantly different in the absence of stage (Figure 1C, *p* = 0.001). Additionally, breast milk and infants’ feces at the same stage showed a different profile of microbiota (Figure 1C, *p* = 0.001).

Bacteria in HBM mainly consisted of *Streptococcus* (BC, 17.2%; BT, 17.0%; BM, 11.6%), *Staphylococcus* (BC, 18.1%; BT, 16.0%; BM, 4.4%), *Acinetobacter* (BC, 2.2%; BT, 18.3%; BM, 14.8%), and *Bifidobacterium* (BC, 11.5%; BT, 9.3%; BM, 18.3%; Figure 1D). *Bifidobacterium* became dominant in the mature milk stage. The microbiota in infants’ feces mainly consisted of *Bifidobacterium* (IC, 43.9%; IT, 54.8%; IM, 51.0%) regardless of stages, whereas *Bacteroides* was dominant in maternal feces (25%, Figure 1E). *Bifidobacterium* in infant feces was significantly higher compared with that in HBM (BC, *p* < 0.05; BT, *p* < 0.01; BM, *p* < 0.01) and maternal feces (IC vs. MF, *p* < 0.05; IT vs. MF, *p* < 0.01; IM vs. MF, *p* < 0.01; Figure 1E). The relative abundances of *Bacteroides*, *Blautia*, and *Faecalibacterium* were significantly higher in maternal feces compared to HBM and infant feces regardless of the stage (*p* < 0.01, Figure 1E). Meanwhile, the *Blautia* of HBM in the colostrum and transition milk stages was significantly higher than that in infant’s feces of the same stage (Figure 1E). However, the relative abundances of *Staphylococcus* and *Streptococcus* in the BC and BT groups were significantly higher compared to those in maternal feces (*p* < 0.05), and *Streptococcus* levels in BC and BM groups were significantly higher than those of the corresponding stage of infant feces (*p* < 0.05, Figure 1F). Furthermore, the relative abundance of *Pseudomonas* in BC and BT groups was significantly higher than that of infant feces (*p* < 0.01), whereas *Veillonella* was significantly lower in BM than that of IM (*p* < 0.01, Figure 1F).

### 2.2. sIgA-Coated and sIgA-Uncoated Bacterial Communities in HBM, Maternal Feces, and Infant Feces

**Figure 2:**
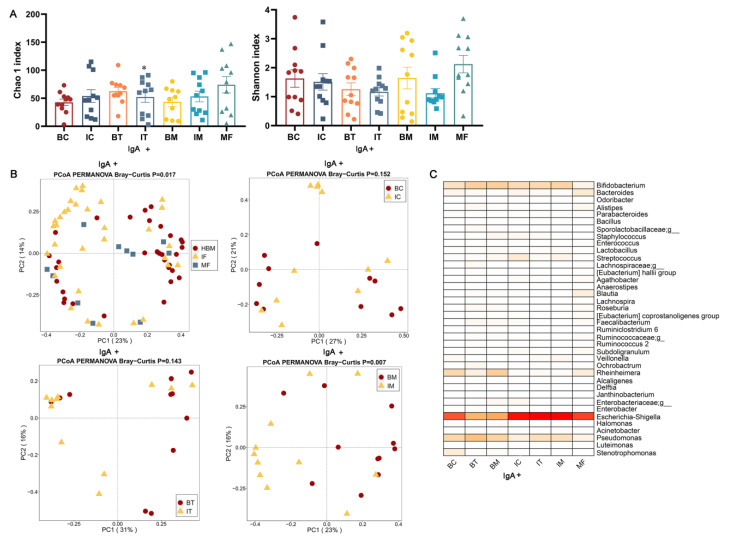
Diversity and composition of sIgA-coated bacteria in HBM, infant feces, and maternal feces. (**A**) Alpha diversity feces. (**B**) Beta diversity. (**C**) Heatmap of the composition of sIgA-coated bacteria in HBM, infant feces, and maternal feces. Genera of relative abundance > 0.01% and calculated with log10 are shown. The darker color corresponds higher relative abundance. The Bray–Curtis distance was utilized to calculate the difference between samples using PERMANOVA. *, *p* < 0.05; three stages of infant feces compared to corresponding HBM. IgA+ means IgA-coated bacteria.

HBM, infant feces, and maternal feces were used to enrich the sIgA-coated and sIgA-uncoated bacteria. sIgA-coated bacteria did not significantly differ in terms of diversity and richness among HBM, maternal feces, and infant feces except for the richness of HBM and infant feces of the transitional stage (Figure 2A). The composition of sIgA-coated bacteria was significantly different among HBM, maternal feces, and infant feces (*p* = 0.017), whereas HBM and infant feces at the same stages only had a significant difference in the composition of sIgA-coated bacteria at the mature milk stage (*p* = 0.007, Figure 2B). The dominant bacterial compositions in sIgA-coated and sIgA-uncoated communities were significantly different from total bacteria. sIgA-coated *Escherichia*-*Shigella* was dominant in HBM, maternal feces, and infant feces, accounting for approximately 50% (BC, 42.9%; BT, 24.8%; BM, 27.9%; IC, 56.6%; IT, 59.6%; IM, 59.4%; MF, 46.6%); in contrast, sIgA-coated *Bifidobacterium* only accounted for 9.3%, 15.7%, and 15.8% in HBM (BC, BT, and BM, respectively); 11.8%, 12.5%, and 14.1% in infant feces (IC, IT, and IM, respectively); and 3.5% in maternal feces (MF, Figure 2C). Additionally, no significant differences were found between sIgA-coated bacteria among the three stages of HBM, maternal feces, and infant feces.

**Figure 3:**
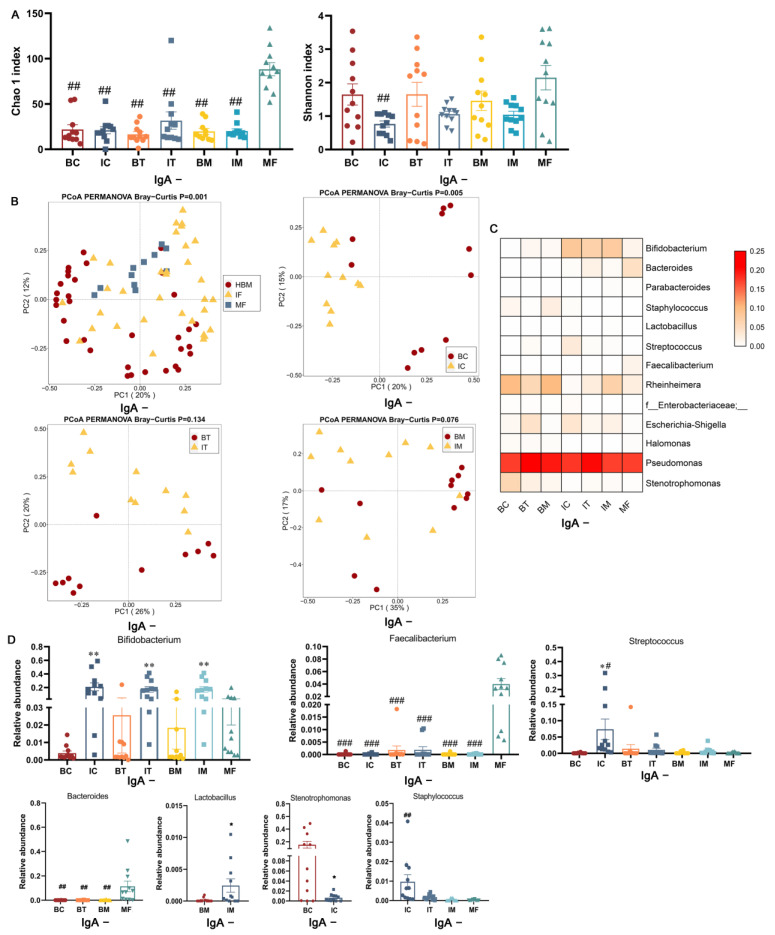
Diversity and composition of sIgA-uncoated bacteria in HBM, infant feces, and maternal feces. (**A**) Alpha diversity. (**B**) Beta diversity. (**C**) Heatmap of the composition of sIgA-uncoated bacteria in HBM, infant feces, and maternal feces. Genera of relative abundances > 0.01% and calculated with log10 are presented. The darker color corresponds to higher relative abundance. The Bray–Curtis distance was utilized to calculate the difference between samples using PERMANOVA. (**D**) The significantly different genera of sIgA-uncoated bacteria between different samples. *, *p* < 0.05; **, *p* < 0.01; three stages of infant feces compared to corresponding BC, BT, and BM. #, *p* < 0.05; ##, *p* < 0.01; ###, *p* < 0.001; three stages of HBM and infant feces compared to maternal feces. BC, BT, BM, IC, IT, and IM stand for colostrum, transitional milk, mature milk, and infant feces corresponding to HBM stages. IgA+ means IgA-coated bacteria, and IgA−means IgA-uncoated bacteria.

The richness of sIgA-uncoated bacteria in maternal feces was significantly higher than HBM and infant feces regardless of the stage (*p* < 0.01, Figure 3A), whereas no significant differences in diversity were observed except for colostrum and the corresponding infant feces, where the latter was significantly lower (*p* < 0.01, Figure 3A). Similar to sIgA-coated bacteria, the composition of sIgA-uncoated bacteria was significantly different among HBM, maternal feces, and infant feces (*p* = 0.001, Figure 3B). In contrast to sIgA-coated bacteria, HBM only showed a significantly different composition of sIgA-uncoated bacteria within the colostrum stage (*p* = 0.005, Figure 3B). sIgA-uncoated bacteria were mainly dominated by *Pseudomonas* in HBM (BC, 49.0%; BT, 61.5%; BM, 56.5%), maternal feces (MF, 48.8%), and infant feces (IC, 50.5%, IT, 59.8%; IM, 48.8%). In addition, sIgA-uncoated *Rheinheimera*, *Bifidobacterium*, and *Bacteroides* were the second-dominant genus in HBM (BC, 25.9%; BT, 14.6%; BM, 26.5%), infant feces (IC, 21.0%, IT, 17.2%; IM, 19.8%), and maternal feces (MF, 11.4%), respectively (Figure 3C). sIgA-uncoated *Bifidobacterium* revealed a higher relative abundance in three stages of infant feces compared to the corresponding HBM (*p* < 0.01, Figure 3D). Maternal feces showed a higher relative abundance of sIgA-uncoated *Faecalibacterium* compared to three stages of HBM and infant feces (*p* < 0.001, Figure 3D) and showed a higher relative abundance of sIgA-uncoated *Bacteroides* compared to HBM (*p* < 0.01, Figure 3D). Interestingly, the relative abundance of sIgA-uncoated *Streptococcus* in infant feces of colostrum stage (IC) was significantly higher compared with colostrum (BC, *p* < 0.05) and maternal feces (MF, *p* < 0.05), and the relative abundance of sIgA-uncoated *Staphylococcus* in infant feces was significantly higher than that of MF (*p* < 0.01, Figure 3D). The relative abundance of sIgA-uncoated *Lactobacillus* only showed a significant difference between HBM and infant feces within the mature stage, being greater in infant feces (*p* < 0.05, Figure 3D), and sIgA-uncoated *Stenotrophomonas* was significantly higher in colostrum compared to the corresponding infant feces (*p* < 0.05, Figure 3D). Interestingly, although the dominant genus of sIgA-coated and -uncoated bacteria in HBM, maternal feces, and infant feces was different, this did not result in a significant difference in the abundance of the genus.

**Figure 4:**
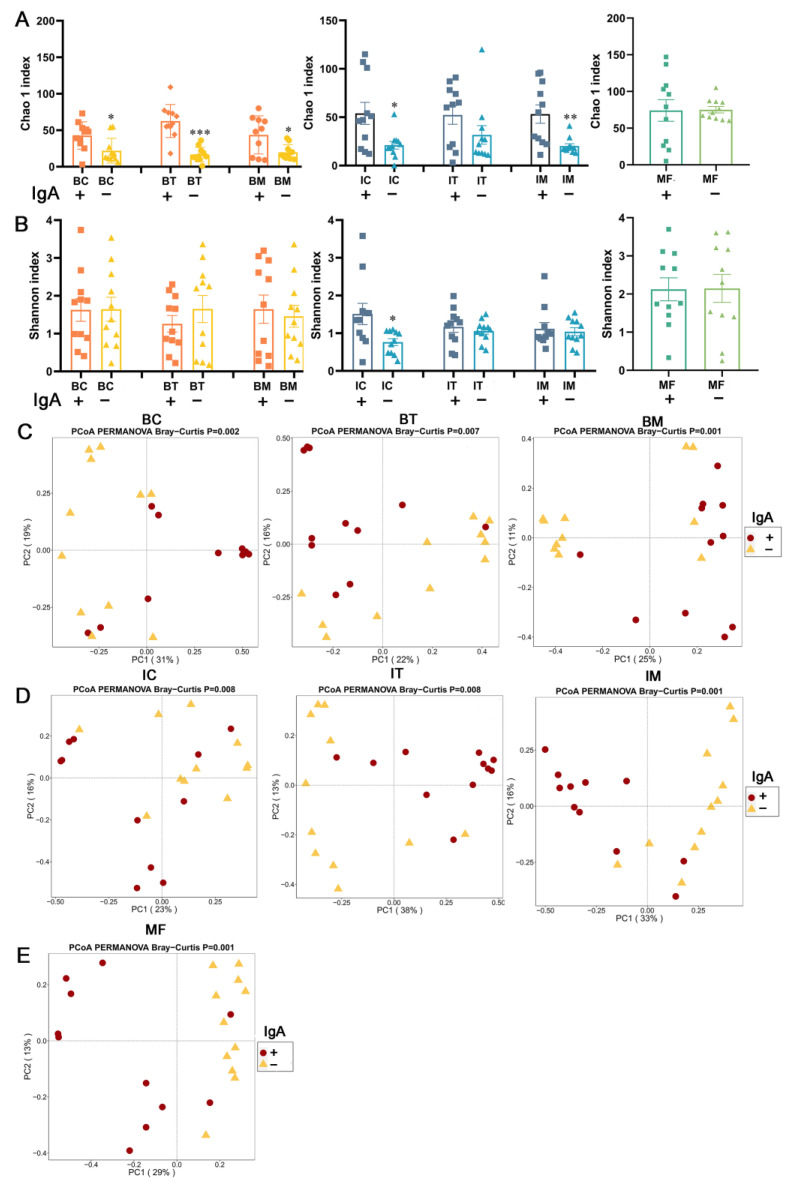
Comparison of diversity between sIgA-coated and -uncoated bacteria in HBM, infant feces and maternal feces. (**A**,**B**) Alpha diversity. (**C**–**E**), Beta diversity The Bray–Curtis distance was utilized to calculate the difference between samples using PERMANOVA. *, *p* < 0.05; **, *p* < 0.01; ***, *p* < 0.001; three stages of infant feces compared to corresponding HBM. BC, BT, BM, IC, IT, and IM stand for colostrum, transitional milk, mature milk, and infant feces corresponding to HBM stages. / means unsorted bacteria, IgA+ means IgA-coated bacteria, and IgA− means IgA-uncoated bacteria.

The alpha diversity was compared between sIgA-coated and sIgA-uncoated bacteria of each sample. Significant lower richness was found in sIgA-uncoated bacteria of the three stages of HBM compared with sIgA-coated bacteria (*p* < 0.05, *p* < 0.001, *p* < 0.05, Figure 4). For infant feces, the richness and diversity of sIgA-uncoated bacteria in the colostrum stage were significantly lower than that of sIgA-coated bacteria (*p* < 0.05, Figure 4A,B). Additionally, the compositions of sIgA-coated and uncoated bacteria were significantly different in HBM (Figure 4C), infant feces (Figure 4D) and maternal feces (Figure 4E).

### 2.3. Total, sIgA-Coated and sIgA-Uncoated Bifidobacterial Community in HBM, Maternal Feces and Infant Feces

Total bifidobacterial composition was analyzed in HBM, infant feces, and maternal feces, whereas sIgA-coated and sIgA-uncoated *Bifidobacterium* communities were only analyzed in infant feces and maternal feces due to low relative abundance in HBM. For infant feces, eight infant feces of colostrum and transition stage, respectively, and six infant feces of mature milk stage were amplified successfully. All maternal feces were amplified successfully.

**Figure 5:**
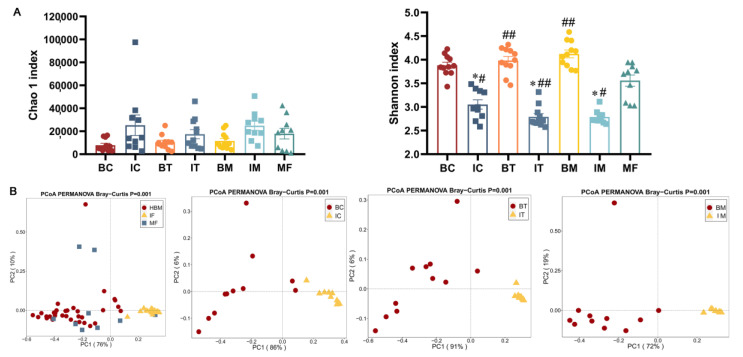
Diversity of total *Bifidobacterium* in HBM, infant and maternal feces. (**A**) Alpha diversity. (**B**) Beta diversity. PERMANOVA was used to calculate differences among samples based on Bray-Curtis distance. *, *p* < 0.05; three stages of infant feces compared to corresponding BC, BT, and BM. #, *p* < 0.05; ##, *p* < 0.01; three stages of HBM and infant feces compared to maternal feces. HBM, IF, MF stand for group HBM, infant and maternal feces. BC, BT, BM, IC, IT, and IM stand for colostrum, transitional milk, mature milk, and infant feces corresponding to HBM stages.

The total *Bifidobacterium* of HBM, maternal feces and infant feces did not show significant differences in richness (Figure 5A). However, the diversity of *Bifidobacterium* in the three stages of infant feces was lower than maternal feces (IC vs. MF, *p* < 0.05; IT vs. MF, *p* < 0.01; IM vs. MF, *p* < 0.05) and the corresponding stage of HBM (*p* < 0.05, Figure 5A). For sIgA-coated and uncoated *Bifidobacterium*, no significant differences were found between maternal and infant feces in richness or diversity (Figure S1A,C). We did not amplify *Bifidobacterium* in HBM due to its low relative abundance at the genus level. Similar to total bacteria, the bifidobacterial compositions in HBM, maternal feces and infant feces were significantly different (*p* = 0.001, Figure 5B). Meanwhile, the composition of Bifidobacterium in breast milk and infant feces at the same stage was significantly different (*p* = 0.001 for three stages, Figure 5B). The composition of sIgA-coated *Bifidobacterium* was similar between maternal and infant feces (*p* = 0.573), whereas sIgA-uncoated bifidobacterial composition was significantly different between maternal and infant feces (*p* = 0.004, Figure S1B,D).

**Figure 6:**
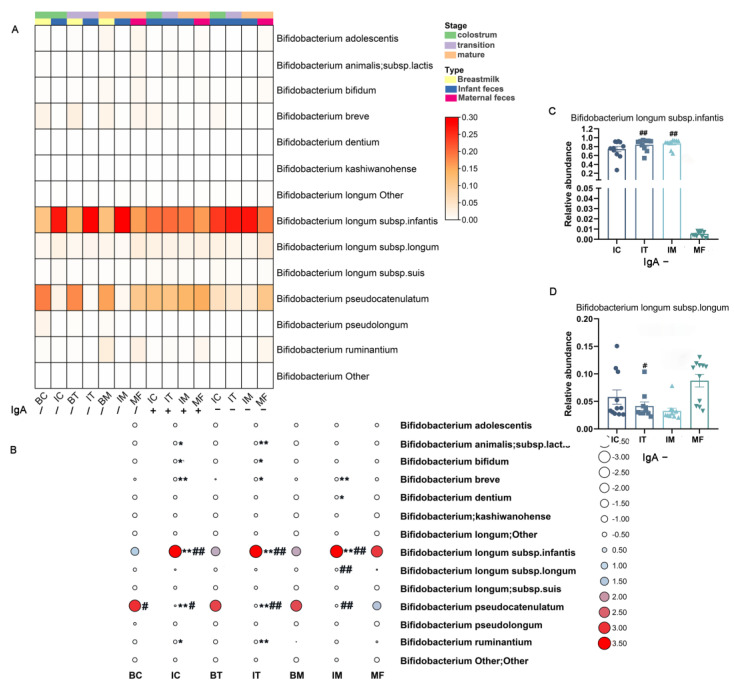
Distribution of *Bifidobacterium* in total sIgA-coated and -uncoated *Bifidobacterium* in HBM, infant feces, and maternal feces. (**A**) Heatmap of the composition of the total sIgA-coated and -uncoated *Bifidobacterium* in HBM, infant feces, and maternal feces. Genera of relative abundances > 0.01% and calculated with log10 are shown. The darker color corresponds to higher relative abundance of the genus. (**B**) The significantly different total *Bifidobacterium* between different samples. The relative abundance of *Bifidobacterium* is shown in column normal normalized. The greater the relative quantity of *Bifidobacterium*, the deeper the red hue and the larger the circle. Greater abundance is indicated by larger white circles. *, *p* < 0.05; **, *p* < 0.01; three stages of infant feces compared to corresponding BC, BT, and BM. (**C**,**D**) Relative abundances of sIgA-uncoated *Bifidobacterium* in infant and maternal fecal samples. #, *p* < 0.05; ##, *p* < 0.01; three stages of HBM and infant feces compared to maternal feces. BC, BT, BM, IC, IT, and IM stand for colostrum, transitional milk, mature milk, and infant feces corresponding to HBM stages. / means unsorted bacteria; IgA− means IgA-uncoated bacteria.

Thirteen bifidobacterial species were detected in our study (Figure 6A). *B. pseudocatenulatum* was dominant in HBM (BC, 53.6; BT, 50.4%; BM, 43.3%), while *B. longum* subsp. *infantis* was dominant in maternal and infant feces (MF, 44.8%; IC, 85.9%; IT, 92.8%; IM, 93.6%, Figure 6A). Similar to total *Bifidobacterium*, sIgA-coated and sIgA-uncoated *B. longum* subsp. *infantis* was dominant both in maternal and infant feces (MF, 45.3%; IC, 56.8%; IT, 59.1%; IM, 54.3%; Figure 6A, MF, 54.2%; IC, 74.4%; IT, 84.0%; IM, 86.9%; Figure 6A).

We then compared the difference of total sIgA-coated and -uncoated *Bifidobacterium* among HBM, maternal feces, and infant feces. Maternal feces showed a significantly lower abundance of *B. longum* subsp. *infantis* compared to that in infant feces (IC, IT, IM, *p* < 0.01, Figure 6B), and maternal feces showed a higher relative abundance of *B. longum* subsp. *longum* compared to infant feces within the mature stage (IM, *p* < 0.01). Additionally, the relative abundance of *B. pseudocatenulatum* was higher in maternal feces compared to that in infant feces (IC, *p* < 0.05; IT, *p* < 0.01; IM, *p* < 0.01) but lower than that in colostrum (BC, *p* < 0.05). Furthermore, when the infant feces with the same stage of HBM from their mother were compared, a higher relative abundance of *B. animalis* subsp. *lactis* and *B. bifidum* and a lower relative abundance of *B. pseudocatenulatum* and *B. ruminantium* were found in infant feces from colostrum and transitional stages compared to HBM (BC vs. IC, *p* < 0.05, *p* < 0.05, *p* < 0.01, *p* < 0.05; BT vs. IT, *p* < 0.01, *p* < 0.05, *p* < 0.01, *p* < 0.01). The three stages of infant feces showed lower relative abundance *B. breve* and higher relative abundance of *B. longum* subsp. *infantis* compared to corresponding HBM (BC vs. IC, *p* < 0.01, *p* < 0.01; BT vs. IT, *p* < 0.05, *p* < 0.01; BM vs. IM, *p* < 0.01, *p* < 0.01; Figure 6B).

For sIgA-coated or sIgA-uncoated *Bifidobacterium*, HBM samples were not amplified successfully due to their low relative abundance; however, infant and maternal feces were amplified successfully for partial samples. Furthermore, for sIgA-uncoated *Bifidobacterium*, a higher relative abundance of *B. longum* subsp. *infantis* and a lower relative abundance of *B. longum* subsp. *longum* were found in infant feces of transitional (*p* < 0.01) and mature stages (*p* < 0.01) compared to the maternal feces. Indeed, sIgA-uncoated *B. longum* subsp. *longum* in infant feces at all stages was significantly lower than that in maternal feces (IT vs. MF, *p* < 0.01; Figure 6C,D).

**Figure 7:**
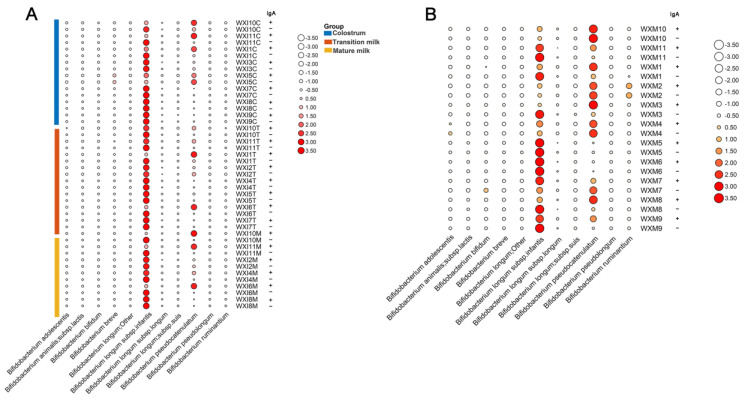
Distribution of sIgA-coated and uncoated *Bifidobacterium* in each sample of infant (**A**) maternal feces (**B**). The relative abundance of *Bifidobacterium* was shown in row normal normalized. The greater the relative quantity of Bifidobacterium, the deeper the red hue and the larger the circle. Greater abundance is indicated by larger white circles. IgA+ means IgA-coated bacteria and IgA− means IgA-uncoated bacteria.

Then, we compared the distribution of sIgA-coated and uncoated *Bifidobacterium* in a single sample of infant feces and maternal feces. In most of the samples from infants and mothers, *B. longum* subsp. *infantis* was the main coated and uncoated *Bifidobacterium* species, while *B. pseudocatenulatum* was the main coated and uncoated *Bifidobacterium* species in a small number of samples (Figure 7). Interestingly, the dominant sIgA-coated *Bifidobacterium* in infant feces at different stages showed stage-dependence. For example, in the feces from infant WXI10 and WXI11, sIgA-coated *B. pseudocatenulatum* was dominant in the colostrum and mature milk stage, while in the transitional stage, the dominant sIgA-coated *Bifidobacterium* species turned to *B. longum* subsp. *infantis* (Figure 7A). Furthermore, the sIgA-coated *Bifidobacterium* in the infant feces was also different from their maternal feces in part of the stage (Figure 7). In addition, similar results were found for sIgA-uncoated *Bifidobacterium* in both maternal feces and infant feces (Figure 7).

### 2.4. Total Lactobacillus Community in Maternal and Infant Feces

**Figure 8:**
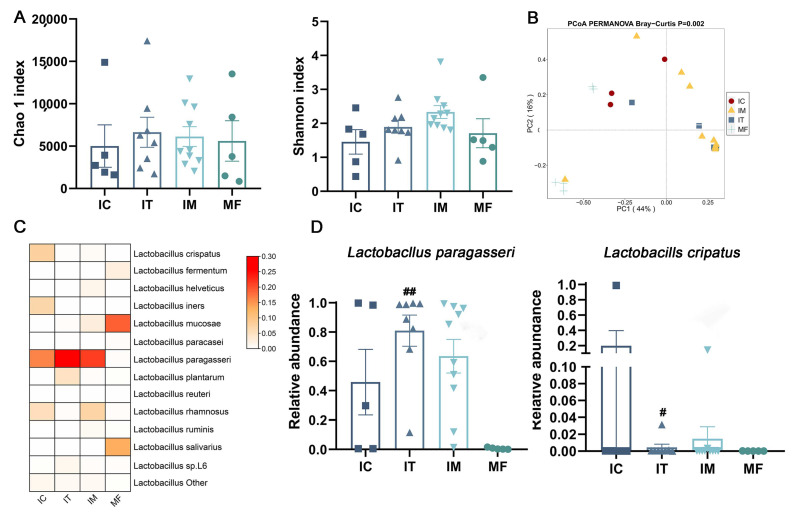
Diversity and composition of *Lactobacillus* in infant and maternal feces. (**A**) Alpha diversity and (**B**) beta diversity. PERMANOVA was used to calculate difference among samples based on Bray–Curtis distance. (**C**) Relative abundance of *Lactobacillus* was calculated with log10. Darker color corresponds to the genus of higher relative abundance. (**D**) Significant differences within *Lactobacillus* species across different samples. #, *p* < 0.05, ##, *p* < 0.01; three stages of infant feces compared to maternal feces. IC, IT, and IM stand for infant feces corresponding to colostrum, transitional milk, and mature milk. MF stands for maternal feces.

The relative abundance of *Lactobacillus* in HBM (BC, BT, and BM) was 2.7%, 0.5%, and 0.1%, respectively, which proved restrictive for further lactobacilli *groEL* sequencing. For fecal samples, we successfully amplified total *Lactobacillus* in a subset of maternal and infant feces successfully (IC, n = 5; IT, n = 8; IM, n = 5; MF, n = 5), and then, the alpha and beta diversity of total *Lactobacillus* was evaluated. No significant difference was found in alpha diversity between the different stages of infant feces or between maternal and infant feces (Figure 8A). However, the composition of *Lactobacillus* among the three stages of infant feces and maternal feces differed significantly (*p* = 0.002, Figure 8B).

We obtained thirteen *Lactobacillus* species in total, while a relative abundance of lower than 0.001% or only detected in a single sample was classified as “*Lactobacillus* other”. *L. paragasseri* was dominant in infant feces in each stage (IC, 45.8%; IT, 81.0%; IM, 63.6%), whereas *L. mucosae* was dominant in maternal feces (MF, 35.5%) (Figure 8C).

Infant feces in the colostrum stage showed a lower relative abundance of *L. mucosae* compared to maternal feces (*p* < 0.01), while those infant feces within transitional and mature stages showed a higher relative abundance of *L. paragasseri* (IT vs. MF, *p* = 0.01; Figure 8D) and *L. crispatus* (IT vs. MF, *p* = 0.05; Figure 8D) compared to maternal feces.

## 3. Discussion

This study compared the composition of bacteria at genus level and bifidobacterial and lactobacilli communities at species level in HBM, maternal feces, and infant feces. Furthermore, the study also investigated sIgA-coated and -uncoated bacteria at genus level and *Bifidobacterium* at species level in the same sample set. Samples from different niches exhibited distinct microbiota profiles in total bacteria, bifidobacterial community, and *Lactobacillus* community as well as in the sIgA-coated and -uncoated bacteria and *Bifidobacterium*. With regard to total bacteria, maternal fecal microbiota showed higher alpha diversity and unique bacterial structure compared to that in HBM and infant feces in line with previous research [19]. Meanwhile, infant feces showed lower richness and diversity compared to that in the same stage of HBM except for the diversity of the colostrum-stage feces that was higher than its HBM counterpart, which may indicate that colostrum consisted of bacteria with low relative abundance. In addition, although a part of infant gut microbiota came from breast milk, the two types of samples showed different microbial compositions in all three stages, similar to previous reports [2,20]. HBM was dominated by *Streptococcus* and *Staphylococcus* within one month of lactation stage, which may mainly come from oral reflux [21] and breast skin contamination [22]. Furthermore, in the mature milk stage, this dominant position was replaced by *Bifidobacterium*. It is well-known that the gut microbiota of breast-fed infants is dominated by *Bifidobacterium* [23] due to its capacity to metabolize human milk oligosaccharides, and this dominance can last for up to half a year after weaning [24]. *Bacteroides* occupies the position of dominant bacterium in the adult gut, and this reshuffling coincides with the introduction of solid food to the infant [25]. Additionally, *Streptococcus* was detected in the infant fecal samples but is known to decrease with age [26,27]. This is consistent with our results of microbial composition in the maternal intestine.

This study also analyzed the composition of the bifidobacterial and lactobacilli communities at the species levels in HBM, maternal feces, and infant feces based on the specific *groEL* genes. Consistent with previous research [28], *B. longum* subsp. *infantis* was the most common *Bifidobacterium* species in infant feces, while HBM and maternal feces were mainly composed of *B. pseudocatenulatum* [28,29]. In contrast, a previous study reported that *B. longum* was common in human breastmilk based on RT-PCR [30]. The profile of *Bifidobacterium* in infant feces and maternal feces was relatively scattered and not concentrated to certain types of *Bifidobacterium* [31].

*Lactobacillus*, which is also necessary for milk digestion, is a commensal microorganism in the infant intestine. It does, however, grow increasingly plentiful after infancy, and its relative abundance declines with age, possibly due to competition with endogenous lactose digesting enzymes in infants [32]. Although the abundance of *Lactobacillus* in infancy increased, it maintained a low abundance level based on both the current study and a previous report [33]. Likewise, the infant gut microbiome included fewer microorganisms that can degrade fiber, including *Ruminococcaceae* and *Lachnospiraceae*. In addition, *L. crispatus* was detected in infant feces at a low relative abundance and was not detected in maternal feces. This suggests that vaginally derived *Lactobacillus* (from vaginal delivery) can survive in the gastrointestinal tract of infants for a short time but might not survive well in the gastrointestinal tract of adults [34,35].

IgA, a critical immunoglobulin in mediating early health in the infant, cannot be synthesized endogenously within the first four weeks after birth and can only be obtained from breast milk [36]. Babies receive 0.25–0.5g/d of immunoglobulin A from breast milk, with colostrum having the highest levels. The amount of IgA in milk decreases with the prolongation of lactation, but the amount of IgA gained by the infant remains roughly the same because of the increase in milk production [36]. Early exposure to passive sIgA in breast milk has a favorable effect on offspring intestinal epithelial cells for the remainder of the life through influencing gene expression in progeny intestinal epithelial cells [37]. Additionally, the specificity of sIgA in breast milk depended on maternal exposure to symbiotic bacteria in the maternal gut. This may allow sIgA to cover enteric bacteria found in breast milk and aid in their colonization of the infant’s gut. Bacterial flow cytometry, together with MACS and high-throughput sequencing approaches, have been used to identify sIgA-coated bacteria [9]. However, we used MACS to focus on separating sIgA-coated and -uncoated bacteria to avoid causing death of some bacteria during the flow cytometry process. Thus, the screening of live *Bifidobacterium* and *Lactobacillus* in further experiments needs to be performed by mining function.

Recent studies have reported that sIgA coats a broad range of bacteria [38], including members of Actinobacteria, Bacteroidetes, Firmicutes, and Proteobacteria [39]. However, it is hard to define a core sIgA-coated bacterial composition in healthy adults due to several factors, such as geographic location, age, and sequencing method [40,41]. Our research found that the composition of sIgA-coated bacteria and -uncoated bacteria from different samples from mother–infant pairs (breast milk, maternal feces, and infant feces) were completely different. It was interesting that the composition of sIgA-coated bacteria in breast milk and infant feces was different in the mature milk stage, and the sIgA-uncoated bacteria differed only in the colostrum period. This seems to confirm that the composition of sIgA conjugated bacteria changes according to the environment, which also explains the difficulty in summarizing the core sIgA-coated bacteria [13]. For example, *Bacteroides*, *Roseburia*, and *Dorea* have been detected in sIgA^+^ form in adults’ feces, whereas, in infant feces, *Bifidobacterium*, *Lactobacillus*, and *Clostridium* were detected as sIgA^+^ [9,15,42], which is partly consistent with our study. Chao1 and Shannon index represent the alpha diversity of bacterial community, respectively. The richness and diversity of fecal microbiota in infants were lower than those in maternal samples due to their single diet, regardless of unsorted, sIgA-coated, and sIgA-coated bacteria. The significant difference in beta diversity of the sIgA-coated and -uncoated bacteria did not result in a difference in the abundance of sIgA-coated bacteria among groups, but the sIgA-uncoated bacteria showed a difference in abundance. At the same time, it was also found that the alpha diversity of sIgA-coated bacteria was higher than that of uncoated bacteria, which could further confirm the results of the previous study that 20–50% of the bacteria in the intestine can be coated by sIgA [43]. In another study using a mouse model, the ratio of sIgA-coated to -uncoated Bacteroidetes and Firmicutes achieved a steady state of roughly 1:1 with age [44].

However, few studies have focused on the profile of sIgA-uncoated bacteria in the human intestine, thus increasing the difficulty for researchers to summarize the variety and the ratio between sIgA-coated and -uncoated bacteria in healthy infants and adults.

A recent study suggested that the occurrence of specific sIgA-coated bacteria in the infant’s gut represented a warning indicator of impending sickness [9]. For example, *Enterobacteriaceae* coated with sIgA increased in the infant’s intestines up to 40 days following diagnosis of necrotizing enterocolitis [45]. In addition, similar results were found in patients with inflammatory bowel disease [11]. Thus, by monitoring changes in the quantity of sIgA-coated bacteria in the gut, it may be possible to accurately forecast the emergence and progression of illnesses [8]. Therefore, an in-depth understanding of the composition of intestinal sIgA-coated bacteria is necessary, especially in relation to the status of the dominant intestinal bacterium, *Bifidobacterium*, in infants.

*Bifidobacterium* is recognized as the dominant bacterium in the infant intestine, especially *B. longum* subsp. *infantis* [23]. *Bifidobacterium* has the ability to induce high levels of IgA production in the intestine, which promotes opportunity for it to be coated with sIgA [46]. *Bifidobacterium* has been shown to be highly coated with sIgA in the infant gut [15]. Some studies have confirmed that sIgA coats *B. longum* [13], which is consistent with our results. In addition, we found that *B. longum* subsp. *infantis* was also the main uncoated bacterium of *B. longum*. Therefore, this result shows that a single bacterial species can exist either in the form of being coated or uncoated by sIgA, as previously shown [47]. Interestingly, we also found that the dominant sIgA-coated *Bifidobacterium* in some samples changed at certain stages. For example, the dominant sIgA-coated *Bifidobacterium* was *B. longum* subsp. *infantis* in the feces of infants in the transition milk stage but, in the same samples, was *B. pseudocatenulatum* in the colostrum and mature milk stages, which is also common in maternal feces in sIgA^+^ form. A study has indicated that after birth, the relative abundance of sIgA-coated *B. longum* reaches the peak within the first six months [13]. Therefore, age is a factor that influences the structure of sIgA-coated bacteria in the human gut [12,13]. Additionally, drug intervention, especially antibiotics, not only impact total bacteria but also influence the composition of sIgA coated bacteria, particularly increasing the relative abundance of sIgA-coated *Lactobacillus* and *Enterococcus* [41]. Hence, the factors that influence the community of sIgA-coated and -uncoated bacteria both at genus and species levels should be further investigated.

## 4. Materials and Methods

### 4.1. Volunteer Recruitment and Sample Collection

Eleven pregnant women were recruited who delivered at the Affiliated Wuxi Maternity and Child Health Care Hospital of Nanjing Medical University from February 2020 to April 2021 (Ethics number: 2020-01-0302-03). All infants were full-term (>38 weeks), vaginally delivered, and breastfed exclusively during the whole sampling collection period. Sample collection included human breast milk (HBM) (n = 33), infant feces (n = 33), and maternal feces (n = 11). HBM samples were collected according to the time after delivery: colostrum (days 1–7), transitional milk (days 8–14), and mature milk (days 15 onwards). The collection time of infant feces was the same as that for breast milk collection, and maternal feces were only collected during the mature milk period. Feces and breast milk sample collection was standardized for all subjects [48]. Samples were stored at −80 °C after collection for further analysis.

### 4.2. Enrichment of sIgA-Coated Bacteria through Magnetic-Activated Cell Sorting

Milk samples were divided into two parts: one (5 mL) for DNA extraction directly and the other (10 mL) for the enrichment of sIgA-coated bacteria; 20 mg fecal samples were used for sIgA-coated bacteria and 50 mg for DNA extraction. Enrichment of sIgA-coated bacteria was performed as previously described [49]. Briefly, bacterial mass was suspended in PBS including 0.5% L-cysteine (PBSL). Next, 500 μL goat serum (Sangon Biotech, Shanghai, China) was added and incubated for 20 min. Then, the bacterial sediment was collected after centrifuging at 6000 rcf, 4 °C, for 5 min. Then, 20 μL IgA antibody (Abcam, Cambridge, UK) and 500 μL carboxyl magnetic beads (Sangon Biotech, Shanghai, China) were added and incubated for 20 min after the bacterial mass suspended in PBSL, respectively. Finally, the bacterial sediment was collected with magnetic rack as sIgA-coated bacteria, and the supernatant was collected as sIgA-uncoated bacteria. Then, sIgA-coated bacteria and sIgA-uncoated bacteria were collected for further DNA extraction, respectively.

### 4.3. DNA Extraction and 16S rRNA Amplification

The FastDNA Spin Kit for Feces (MP Biomedicals, LLC, Irvine, CA, USA) was used for DNA extraction. The PCR reaction was performed as previously described [50]. Bacterial sediments of HBM, infant feces, and maternal feces were deposited in Lysing. Each matrix E tube was filled with 825 μL sodium phosphate buffer and 275 μL PLS dissolving solution and vortexed for 10–15 s before centrifugation at 14,000× *g* for 5 min, afterwards discarding the supernatant. Following that, 978 μL sodium phosphate buffer and 122 μL MT buffer were added, and the mixture was agitated and then broken for 30 s (35 times) at 70 HZ on the high-throughput tissue grinder. The Lysing Matrix E tube was then centrifuged for 10 min at 14,000× *g*. Finally, bacterial DNA was extracted from the supernatant using the FastDNA Spin Kit for Feces. PCR amplification for the V3–V4 region of the 16S rRNA gene was conducted as previously described [51]. The primers were as follows: 341F:5′-CCT AYG GGRBGCASCAG-3′ and 806R:5′-GGA CTA CNNGGG TAT CTAAT-3′. Negative controls were included using deionized sterile water as template.

### 4.4. Bifidobacteria and Lactobacilli groEL Gene Amplification

The procedure for the amplification of the *Bifidobacterium* and *Lactobacillus groEL* genes was the same as for the 16S rRNA gene, but specific primer pairs for bifidobacterial and lactobacilli *groEL* genes were used [52]. The *Bifidobacterium* *groEL* gene was amplified using the primers Bif-*groEL*-F (5′-TCCGATTACGAYCGYGAGAAGCT-3′)/Bif-*groEL*-R (5′-CSG CYTCGGTSGTCAGGA-ACA-G-3′), and the *Lactobacillus* gene was amplified using primers Lac-*groEL*-F (5′-GCYGGTG-CWAACCCNGTTGG-3′)/Lac-*groEL*-R (AANGTNCCVCGVATC-TTGTT-3′) to differentiate the species. Negative controls using deionized sterile water as the template were included.

### 4.5. Illumina MiSeq Sequencing

The PCR products (465 bp for the 16S rRNA V3-V4 region and 480 bp for the *groEL* genes) were extracted from a 1.5 percent agarose gel and purified using the QIAquick Gel Extraction Kit (Biomiga, Hangzhou, China) and quantified with the QubitTM dsDNA BR Assay Kit (Thermofisher, Waltham, MA, USA) according to the manufacturer’s instructions. The TruSeq DNA LT Sample Preparation Kit (Illumina, San Diego, CA, USA) was used to build libraries of the 16S rRNA gene, the Bifidobacterium, and Lactobacillus *groEL* genes. These were then sequenced on an Illumina MiSeq sequencer using the MiSeq v3 Reagent Kit (Illumina, San Diego, CA, USA) (600 cycles-PE) according to the manufacturer’s instructions.

### 4.6. Statistical Analysis

To analyze the 16S rRNA sequencing data, the QIIME2 pipeline and DADA2 were used to demultiplex and filter raw sequencing data. Samples were identified by the barcodes attached to 341F, Bif-*groEL*-F, and Lac-*groEL*-F, respectively. The forward and reverse reads were merged and allocated based on the barcode, which was cut off before being aligned with the SILVA database. Sequences were grouped into OTUs after removing chimeric sequences. Sequence similarity greater than 97% was classified as an OTU. Alpha diversity was calculated in QIIME2 including Chao1 and Shannon indexes. Sample resampling depth (generally the minimum sample data amount or the data amount covering the vast majority of samples) was determined by “Table.Qzv” calculated by QIIME2. Beta diversity was calculated using PCoA based on the distance between the Bray–Curtis matrix. Furthermore, *t*-test and ANOVA were used to calculate the difference(s) between two groups and more than two groups, respectively. The data were provided as mean standard error of the mean (SEM). SPSS 25.0 (Stanford, CA, USA) was used to perform statistical testing. The data in Figure 1B,E,F, Figure 2A,D, Figure 3A,D, Figure 4A,D, Figure 5A, Figure 6C,D, Figure 8A,D and Figure S1A,C were all were tested for normal distribution before comparison, and *t*-test was used to determine whether there was a significant difference between the two groups (BC vs. IC, BT vs. IT, BM vs. IM). To compare the differences between groups of more than two, one-way ANOVA was utilized. The Shapiro–Wilk test was used to check for normality and homoscedasticity in the data (by the Levene test). One-way analysis of variance was carried out when the data fulfilled the normal distribution criterion. Post hoc Tukey’s test was used to evaluate the significant difference among groups. The data were analyzed using the Kruskal–Wallis test to compare medians between groups if the differences were statistically significant.

## 5. Conclusions

HBM, infant feces, and maternal feces exhibited a unique diversity and composition of sIgA-coated and -uncoated bacteria at genus level while showing similar dominant sIgA-coated bacteria, namely *Escherichia-shigella*, and dominant uncoated bacteria, namely *Pseudomonas*, respectively. For the bifidobacterial community at species level, infant and maternal feces showed a similar diversity and composition of sIgA-coated and -uncoated *Bifidobacterium*, whereas *B. longum* subsp. *infantis* was dominant in infant feces, and *B. pseudocatenulatum* was dominant in maternal feces either in sIgA-coated or -uncoated form. In addition, even a single *Bifidobacterium* species could be found both in sIgA-coated and -uncoated form.

## Data Availability

Not applicable.

## Supplementary Materials

The following supporting information can be downloaded at: https://www.mdpi.com/article/10.3390/ijms23179873/s1.

## Abbreviations

HBM, human breast milk; IF, infant feces; MF, maternal feces; BC, colostrum; BT, transitional breastmilk; BM, mature breastmilk; IC, infant feces corresponding to stage of colostrum; IT, infant feces corresponding to stage of transitional breastmilk; IM, infant feces corresponding to stage of mature breastmilk.

## References

[1] Nyangahu D.D., Jaspan H.B. (2019). Influence of maternal microbiota during pregnancy on infant immunity. Clin. Exp. Immunol..

[2] Ferretti P., Pasolli E., Tett A., Asnicar F., Gorfer V., Fedi S., Armanini F., Truong D.T., Manara S., Zolfo M. (2018). Mother-to-infant microbial transmission from different body sites shapes the developing infant gut microbiome. Cell Host Microbe.

[3] Pannaraj P.S., Li F., Cerini C., Bender J.M., Yang S., Rollie A., Adisetiyo H., Zabih S., Lincez P.J., Bittinger K. (2017). Association between breast milk bacterial communities and establishment and development of the infant gut microbiome. JAMA Pediatr..

[4] Baumann J., Park C.G., Mantis N.J. (2010). Recognition of secretory IgA by DC-SIGN: Implications for immune surveillance in the intestine. Immunol. Lett..

[5] Macpherson A.J., Uhr T. (2004). Induction of protective IgA by intestinal dendritic cells carrying commensal bacteria. Science.

[6] Rodriguze J.M. (2014). The origin of human milk bacteria: Is there a bacterial entero-mammary pathway during late pregnancy and lactation?. Adv. Nutr..

[7] Donaldson G.P., Ladinsky M.S., Yu K.B., Sanders J.G., Yoo B.B., Chou W.-C., Conner M.E., Earl A.M., Knight R., Bjorkman P.J. (2018). Gut microbiota utilize immunoglobulin A for mucosal colonization. Science.

[8] Ding M., Yang B., Ross P.R., Standon C., Zhao J., Zhao H., Chen W. (2021). Crosstalk between sIgA-coated bacteria in infant gut and early-life health. Trends Microbiol..

[9] Palm N.W., Zoete M.R.D., Cullen T.W., Barry N.A., Stefanowski J., Hao L., Degnan P.H., Hu J., Peter I., Zhang W. (2014). Immunoglobulin A coating identifies colitogenic bacteria in inflammatory bowel disease. Cell.

[10] Gopalakrishna K.P., Macadangdang B.R., Rogers M.B., Tometich J.T., Firek B.A., Baker R., Ji J., Burr A.H.P., Ma C., Good M. (2019). Maternal IgA protects against the development of necrotizing enterocolitis in preterm infants. Nat. Med..

[11] Lin R.T., Chen H.W., Shu W.G., Sun M., Fang L., Shi Y., Pang Z., Wu W., Liu Z. (2018). Clinical significance of soluble immunoglobulins A and G and their coated bacteria in feces of patients with inflammatory bowel disease. J. Transl. Med..

[12] Dzidic M., Abrahamsson T.R., Artacho A., Björkstén B., Collado M.C., Mira A., Jenmalm M.C. (2017). Aberrant IgA responses to the gut microbiota during infancy precede asthma and allergy development. J. Allergy Clin. Immunol..

[13] Planer J.D., Peng Y., Kau A.L., Blanton L.V., Ndao I.M., Tarr P.I., Warner B.B., Gordon J.I. (2016). Development of the gut microbiota and mucosal IgA responses in twins and gnotobiotic mice. Nature.

[14] Janzon A., Goodrich J.K., Koren O., Waters J.L., Ley R.E., The TEDDY Study Group (2019). Interactions between the gut microbiome and mucosal immunoglobulins A, M, and G in the developing infant gut. mSystems.

[15] Fadlallah J., Kafsi H.E., Sterlin D., Juste C., Parizot C., Dorgham K., Autaa G., Gouas D., Almeida M., Lepage P. (2018). Microbial ecology perturbation in human IgA deficiency. Sci. Transl. Med..

[16] D’Auria G., Peris-Bondia F., Džunková M., Mira A., Collado M.C., Latorre A., Moya A. (2013). Active and secreted IgA-coated bacterial fractions from the human gut reveal an under-represented microbiota core. Sci. Rep..

[17] Dzidic M., Mira A., Alejandro A., Abrahamsson T.R., Jenmalm M.C., Collado M.C. (2019). Allergy development is associated with consumption of breastmilk with a reduced microbial richness in the first month of life. Pediatr. Allergy Immunol..

[18] Meyer K.M., Prince A., Boggan B., Aagaard K.M. (2019). Maternal IgA targets commensal microbiota in breast milk and the maternal and infant gut microbiomes. Am. J. Obstet. Gynecol..

[19] Murphy K., Curley D., O’Callaghan T.F., O’Shea C.-A., Dempsey E.M., O’Toole P., Ross R., Ryan C.A., Stanton C. (2017). The composition of human milk and infant faecal microbiota over the first three months of Life: A Pilot Study. Sci. Rep..

[20] Williams J.E., Carrothers J.M., Lackey K.A., Beatty N.F., Brooker S.L., Peterson H.K., Steinkamp K.M., A York M., Shafii B., Price W.J. (2019). Strong multivariate relations exist among milk, oral, and fecal microbiomes in mother-infant dyads during the first six months postpartum. J. Nutr..

[21] Biagi E., Quercia S., Aceti A., Beghetti I., Rampelli S., Turroni S., Faldella G., Candela M., Brigidi P., Corvaglia L. (2017). The bacterial ecosystem of mother’s milk and infant’s mouth and gut. Front. Microbiol..

[22] Fitzstevens J.L., Smith K.C., Hagadorn J.I., Caimano M.J., Matson A.P., Brownell E.A. (2016). Systematic review of the human milk microbiota. Nutr. Clin. Pract..

[23] Princisval L., Rebelo F., Williams B.L., Coimbra A.C., Crovesy L., Ferreira A.L., Kac G. (2021). Association between the mode of delivery and infant gut microbiota composition up to 6 months of age: A systematic literature review considering the role of breastfeeding. Nutr. Rev..

[24] Bäckhed F., Roswall J., Peng Y., Feng Q., Jia H., Kovatcheva-Datchary P., Li Y., Xia Y., Xie H., Zhong H. (2015). Dynamics and stabilization of the human gut microbiome during the first year of life. Cell Host Microbe.

[25] Prendergast A.J., Humphrey J.H. (2014). The stunting syndrome in developing countries. Paediatr. Int. Child Health.

[26] Dzidic M., Collado M.C., Abrahamsson T., Artacho A., Stensson M., Jenmalm M.C., Mira A. (2018). Oral microbiome development during childhood: An ecological succession influenced by postnatal factors and associated with tooth decay. ISME J..

[27] Cephas K.D., Kim J., Mathai R.A., Barry K.A., Dowd S.E., Meline B.S., Swanson K.S. (2011). Comparative analysis of salivary bacterial microbiome diversity in edentulous infants and their mothers or primary care givers using pyrosequencing. PLoS ONE.

[28] Underwood M.A., German J.B., Lebrilla C.B., Mills D.A. (2015). *Bifidobacterium longum* subspecies *infantis*: Champion colonizer of the infant gut. Pediatr. Res..

[29] Yang B., Yan S., Chen Y., Ross R.P., Stanton C., Zhao J., Zhang H., Chen W. (2020). Diversity of gut microbiota and bifidobacterial community of chinese subjects of different ages and from different regions. Microorganisms.

[30] Gueimonde M., Laitinen K., Salminen S., Isolauri E. (2007). Breast Milk: A source of *Bifidobacteria* for infant gut development and maturation?. Neonatology.

[31] Yang B., Chen Y., Stanton C., Ross R.P., Lee Y.-K., Zhao J., Zhang H., Chen W. (2019). *Bifidobacterium* and *Lactobacillus* composition at species level and gut microbiota diversity in infants before 6 weeks. Int. J. Mol. Sci..

[32] Wang Y.X., Harvey C.B., Hollox E.J., Phillips A.D., Poulter M., Clay P., Walker-Smith J.A., Swallow D.M. (1998). The genetically programmed down-regulation of lactase in children. Gastroenterology.

[33] Zhang X., Mushajiang S., Luo B., Tian F., Ni Y., Yan W. (2020). The composition and concordance of *Lactobacillus* populations of infant gut and the corresponding breast-milk and maternal gut. Front. Microbiol..

[34] Gregorio D., Parolin R.P., Abruzzo A., Luppi B., Protti M., Mercolini L., Silva J.A., Giordani B., Marangoni A., Nader-Macías M.E.F. (2020). Biosurfactant from vaginal *Lactobacillus crispatus* BC1 as a promising agent to interfere with *Candida* adhesion. Microb. Cell Fact..

[35] Kirtzalidou E., Pramaterftaki P., Kotsou M., Kyriacou A. (2011). Screening for lactobacilli with probiotic properties in the infant gut microbiota. Anaerobe.

[36] Rognum T.O., Thrane P.S., Stoltenberg L., Brandtzaeg P. (1992). Development of intestinal mucosal immunity in fetal life and the first postnatal months. Pediatr. Res..

[37] Rogier E.W., Frantz A.L., Bruno M.E.C., Wedlund L., Cohen D.A., Stromberg A.J., Kaetzel C.S. (2014). Secretory antibodies in breast milk promote long-term intestinal homeostasis by regulating the gut microbiota and host gene expression. Proc. Natl. Acad. Sci. USA.

[38] Bunker J.J., Erickson S.A., Flynn T.M., Henry C., Koval J.C., Meisel M., Jabri B., Antonopoulos D.A., Wilson P.C., Bendelac A. (2017). Natural polyreactive IgA antibodies coat the intestinal microbiota. Science.

[39] Sterlin D., Fadlalah J., Slack E., Gorochov G. (2020). The antibody/microbiota interface in health and disease. Mucosal Immunol..

[40] De Filippo C., Cavalieri D., Di Paola M., Ramazzotti M., Poullet J.B., Massart S., Collini S., Pieraccini G., Lionetti P. (2010). Impact of diet in shaping gut microbiota revealed by a comparative study in children from Europe and rural Africa. Proc. Natl. Acad. Sci. USA.

[41] Džunková M., Moya A., Vázquez-Castellanos F., Artacho A., Chen X., Kelly C., D’Auria G. (2016). Active and secretory IgA-coated bacterial fractions elucidate dysbiosis in *Clostridium difficile* infection. mSphere.

[42] Magria G., Comerma L., Pybus M., Sintes J., Lligé D., Segura-Garzón D., Bascones S., Yeste A., Grasset E.K., Gutzeit C. (2017). Human secretory IgM emerges from plasma cells clonally related to gut memory B cells and targets highly diverse commensals. Immunity.

[43] Mathias A., Corthésy B. (2011). Recognition of gram-positive intestinal bacteria by hybridoma- and colostrum-derived secretory immunoglobulin A is mediated by carbohydrates. Biol. Chem..

[44] Mirpuri J., Raetz M., Sturge C.R., Wilhelm C.L., Benson A., Savani R.C., Hooper L.V., Yarovinsky F. (2014). Proteobacteria-specific IgA regulates maturation of the intestinal microbiota. Gut Microbes.

[45] Hackam D., Caplan M. (2018). Necrotizing enterocolitis: Pathophysiology from a historical context. Semin. Pediatr. Surg..

[46] Yasui H., Nagaoka A., Mike K., Hayakawa K., Ohwaki M. (1992). Detection of *Bifidobacterium* strains that induce large quantities of IgA. Microb. Ecol. Health Dis..

[47] Macpherson A.J., Köller Y., McCoy K.D. (2015). The bilateral responsiveness between intestinal microbes and IgA. Trends Immunol..

[48] Albesharat R., Ehrmann M.A., Korakli M., Yazaji S., Vogel R.F. (2011). Phenotypic and genotypic analyses of lactic acid bacteria in local fermented food, breast milk and faeces of mothers and their babies. Syst. Appl. Microbiol..

[49] Sun J., Qi C., Zhu H., Zhou Q., Xiao H., Le G., Chen D. (2019). IgA-Targeted *Lactobacillus jensenii* modulated gut barrier and microbiota in high-fat diet-fed mice. Front. Microbiol..

[50] Ding M., Qi C., Yang Z., Jiang S., Bi Y., Lai J., Sun J. (2019). Geographical location specific composition of cultured microbiota and *Lactobacillus* occurrence in human breast milk in China. Food Funct..

[51] Yan S., Yang B., Zhao J.C., Zhao J.X., Stanton C., Ross R.P., Zhang H., Chen W. (2019). A ropy exopolysaccharide producing strain *Bifidobacterium longum* subsp. *longum* YS108R alleviates DSS-induced colitis by maintenance of the mucosal barrier and gut microbiota modulation. Food Funct..

[52] Yang B., Ding M., Chen Y., Han F., Yang C., Zhao J., Malard P., Stanton C., Ross R.P., Zhang H. (2021). Development of gut microbiota and bifidobacterial communities of neonates in the first 6 weeks and their inheritance from mother. Gut Microbes.

