# Supplementary material for: Shared and Non-Shared sIgA-Coated and -Uncoated Bacteria in Intestine of Mother–Infant Pairs
Source: Int J Mol Sci. 2022 Aug 30;23(17):9873. doi: 10.3390/ijms23179873 (PMC9456154; doi:10.3390/ijms23179873)

Figure S1. Diversity of sIgA-coated and uncoated *Bifidobacterium* in HBM, infant and maternal feces. A, Alpha diversity of sIgA-coated bacteria. B, Alpha diversity of sIgA-uncoated bacteria. C, Beta diversity of sIgA-coated bacteria. D, Beta diversity of sIgA-uncoated bacteria. PERMANOVA was used to calculate differences among samples based on Bray-Curtis distance. *, $p < 0.05$, three stages of infant feces compared to corresponding BC, BT, and BM. #, $p < 0.05$, ##, $p < 0.01$, three stages of HBM and infant feces compared to maternal feces. HBM, IF, MF stand for group HBM, infant and maternal feces. BC, BT, BM, IC, IT, and IM stand for colostrum, transitional milk, mature milk and infant feces corresponding to HBM stages. IgA+ means IgAcoated bacteria, and IgA- means IgA−uncoated bacteria.

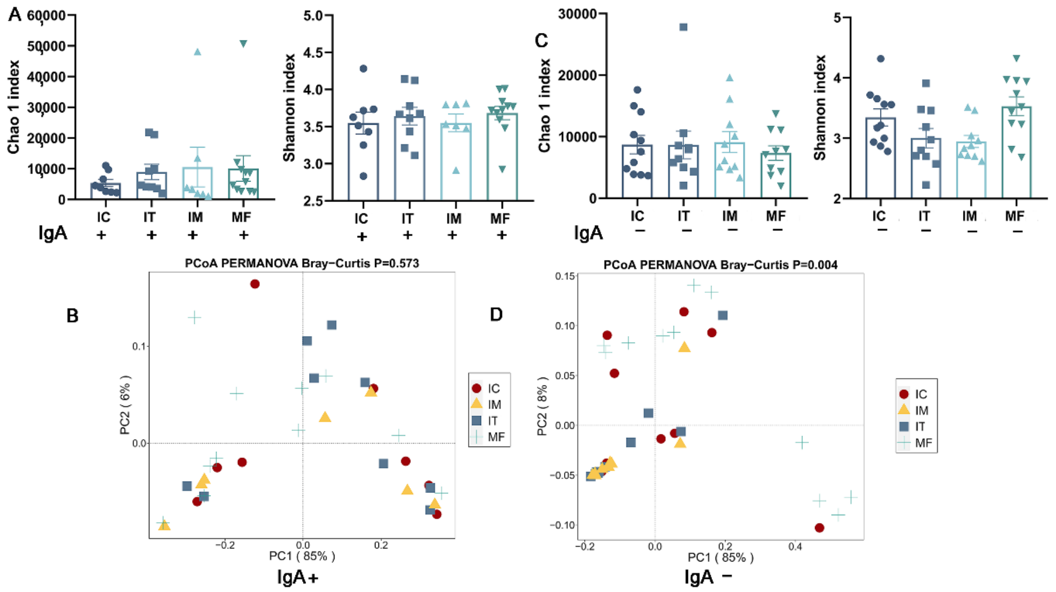

Supplement: Supplementary file 1 [file ijms-23-09873-s001.zip › ijms-1847955-supplementary.pdf]
